# Tumor‐derived exosomes (TDEs): How to avoid the sting in the tail

**DOI:** 10.1002/med.21623

**Published:** 2019-07-18

**Authors:** MeiHua Wan, Bo Ning, Sarah Spiegel, Christopher J. Lyon, Tony Y. Hu

**Affiliations:** ^1^ Department of Integrated Traditional Chinese and Western Medicine West China Hospital of Sichuan University Chengdu Sichuan China; ^2^ Center for Molecular Design and Biomimetics, The Biodesign Institute Arizona State University Tempe Arizona; ^3^ School of Biological and Health Systems Engineering Arizona State University Tempe Arizona; ^4^ Virginia G. Piper Biodesign Center for Personalized Diagnostics, The Biodesign Institute Arizona State University Tempe Arizona

**Keywords:** cancer biomarker, immune escape, immune suppression, tumor‐derived exosomes

## Abstract

Exosomes are abundantly secreted extracellular vesicles that accumulate in the circulation and are of great interest for disease diagnosis and evaluation since their contents reflects the phenotype of their cell of origin. Tumor‐derived exosomes (TDEs) are of particular interest for cancer diagnosis and therapy, since most tumor demonstrate highly elevated exosome secretion rates and provide specific information about the genotype of a tumor and its response to treatment. TDEs also contain regulatory factors that can alter the phenotypes of local and distant tissue sites and alter immune cell functions to promote tumor progression. The abundance, information content, regulatory potential, in vivo half‐life, and physical durability of exosomes suggest that TDEs may represent a superior source of diagnostic biomarkers and treatment targets than other materials currently under investigation. This review will summarize current information on mechanisms that may differentially regulate TDE biogenesis, TDE effects on the immune system that promote tumor survival, growth, and metastasis, and new approaches understudy to counteract or utilize TDE properties in cancer therapies.

## 1 INTRODUCTION

Most cell types secrete extracellular vesicles (EVs) that are involved in intracellular communication, but the EV designation covers a heterogeneous population of vesicles types, including exosomes, microvesicles, and apoptotic bodies, which employ different biogenesis mechanisms.^1^ Exosomes are small (30‐100 nm) vesicles and their biogenesis requires the selective accumulation of cytosolic factors on the endosomal membrane to regulate invagination of the endosome lipid bilayer into its luminal space to produce an intraluminal vesicle (ILV).^2^ Repetition of this process produces a multivesicular body (MVB) filled with ILVs, which can then fuse with the cell membrane through an adenosine triphosphate (ATP)‐dependent process to release exosomes into the adjacent extracellular space.^3^ Exosomes are thus distinct from microvesicles (100‐1000 nm) and apoptotic bodies (100‐5000 nm) that form by outward budding of the plasma membrane, although the lack of size separation between these three groups and overlaps among their membrane proteins complicate the isolation of specific EV types, and can thus negatively impact their utility for diagnostic assays.

Exosomes are the only EVs that are formed by the endocytic pathway and contain factors derived from the cytosol as well as both plasma and endosomal membranes, although this there is a substantial degree of overlap between factors present in exosomes and other EVs.^4^ Exosome carry an array of lipids, proteins, DNA, and RNA (messenger RNAs [mRNAs], microRNAs (miRNAs), and long noncoding RNA [lncRNAs]) that can reflect the phenotype of their cell of origin or altered sorting processes specific to these cells.^5^ Exosomes thus contain a broad array of potential biomarkers that may be useful for early disease diagnosis, the evaluation of pathogen load or disease stage, and monitoring the response of these diseases to treatment. Further, due to their restricted size range, exosomes may be less sensitive to shear forces and more easily transit interstitial space than larger EVs, enhancing their entry into the circulation.

Exosomes are also highly abundant in the circulation and may increase with cancer and other inflammatory conditions,^6^ although it is difficult to specify precise concentration ranges associated with healthy and disease states due to the yield and purity variability inherent in exosome isolation procedures. Measurement of circulating exosome concentrations could theoretically be employed as surrogate diagnostic or prognostic biomarkers of cancer, but such approaches may be subject to significant confounding effects arising from variable exosome secretion from malignant and nonmalignant tissue. More promising efforts have instead focused on analyzing exosome‐associated factors that reveal differential expression between exosomes derived from malignant and nonmalignant cells and tissues. Potential TDE biomarkers include factors contained within the exosome lumen (DNAs, miRNAs lncRNAs, circular RNAs, mRNAs, and proteins), all of which can change with cell stress conditions, including those associated with tumor development or progression.^7^

Several features associated with TDE biogenesis and secretion increase their utility as diagnostic or prognostic biomarkers. Cancer cells exhibit greater exosome secretion rates than nonmalignant cells of the same cell type,^8^ and elevated exosome levels have been reported to distinguish subjects with and without cancer and those who exhibit short versus long survival times.^9^, ^10^ Diagnostic and prognostic judgements based on total exosome abundance are susceptible to confounding effects from altered exosome production by nonmalignant cells, however, since most cells secrete exosomes and secretion rates can be influenced by a variety of conditions. For example, a substantial fraction of the circulating exosome population is derived from platelets and megakaryocytes, and the relative abundance of this exosome population can increase under certain disease conditions.^11^ Direct measurement and analysis of TDEs is more accurate but also faces significant technical challenges. Specific biomarkers are required to differentiate TDEs from exosomes derived from nonmalignant tissues, but while numerous groups have conducted EV biomarker discovery studies to identify cancer‐associated EV factors,^12^ very few EV biomarkers have been subjected to validation studies, and no EV biomarkers have received FDA approval and entered clinical practice.

TDEs, however, are also known to exhibit significant negative regulatory effects on the immune system that can promote immune tolerance and tumor escape from immune surveillance and there has been considerable research focused on this topic. This review will therefore focus on mechanisms that may regulate enhanced exosome production, how these are altered in tumor cells, regulatory effects of TDEs on the immune system, and the status of approaches to directly or indirectly utilize TDEs in new cancer therapeutics that are currently in development.

### 1.1 MVB biogenesis, intracellular transport, and exosome secretion

**Figure 1:**
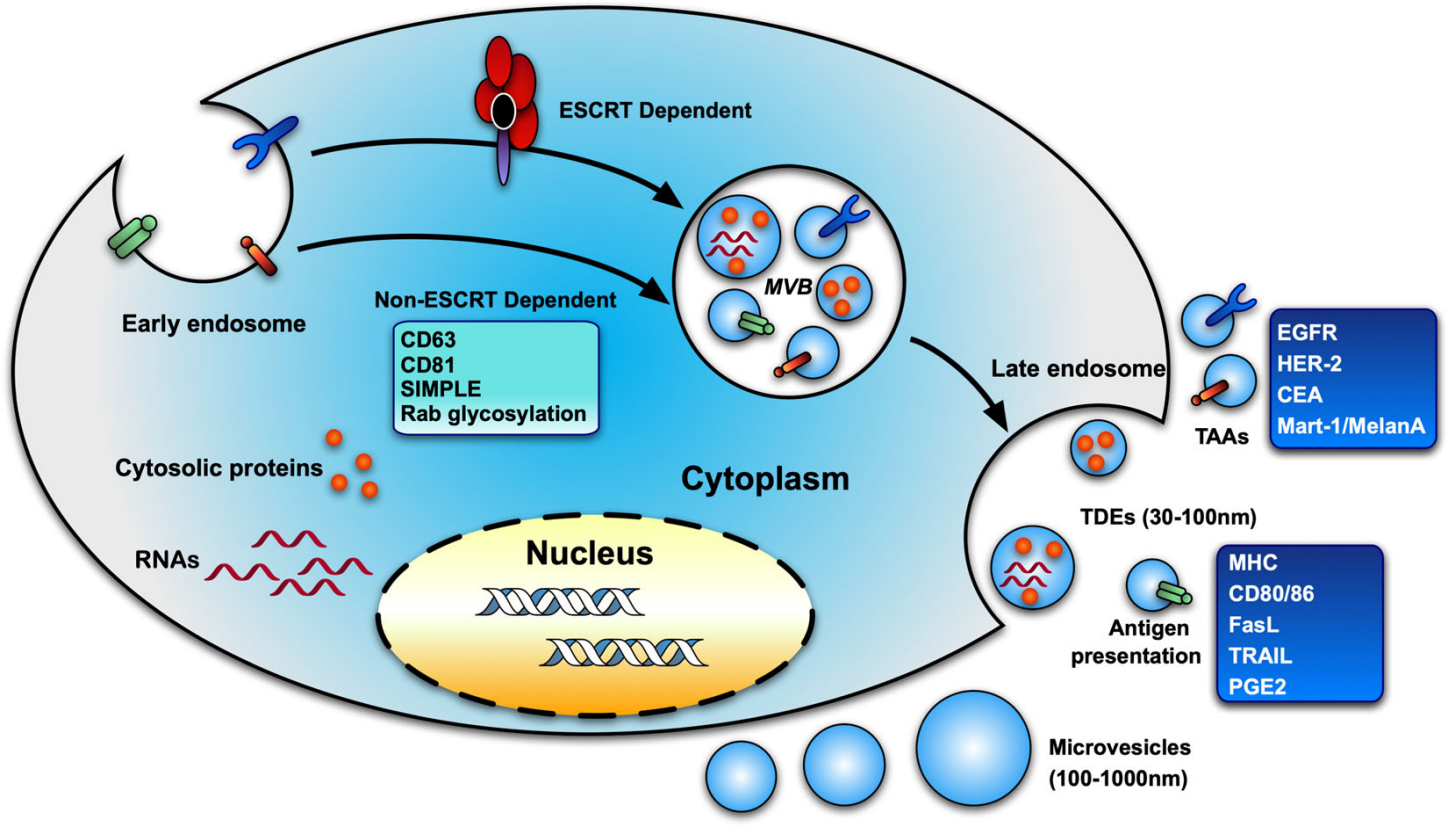
Exosomes (30‐150 nm) form in process where the endosome membrane buds inward to form intraluminal vesicles (ILVs) that accumulate to generate multivesicular bodies (MVBs), which are subsequently released into the extracellular space as mature endosomes. ILV formation is regulated by an endosomal sorting complex required for transport (ESCRT)‐dependent mechanism, as well as non‐ESCRT‐dependent mechanisms mediated by CD63, CD81, SIMPLE, and RAB proteins and RAB‐specific glycosylation events. TDEs can package tumor‐associated antigens (TAAs; eg, epidermal growth factor receptor [EGFR], human epidermal growth factor receptor 2 [HER‐2], carcinoembryonic antigen [CEA], and Mart‐1/MelanA) as well as proteins involved in antigen‐presentation (major histocompatibility complex I [MHC‐I] and MHC‐II), MHC costimulatory molecules (CD80/CD86), death receptor ligands (Fas ligand [FasL] and tumor necrosis factor‐related apoptosis‐inducing ligand [TRAIL]) and inhibitory factors (PGE2). Exosomes (30‐150 nm) partially overlap in size with microvesicles (100‐1000 nm), which bud directly from the plasma membrane, which can complicate the isolation of exosome samples by their physical properties [Color figure can be viewed at wileyonlinelibrary.com]

Exosome secretion requires three distinct processes: the accumulation of ILVs to produce MVB, the migration of these ILV‐rich MVBs to the plasma membrane (or to lysosomes for degradation), and the fusion of MVBs with the plasma membrane to allow the release of their contents into the extracellular space as mature exosomes (Figure 1). Exosome formation appears to be primarily regulated by the lipid composition of the outer leaflet of the endosome membrane and the activity of several proteins complexes that belong to the endosomal sorting complex required for transport (ESCRT) machinery, although studies indicate that there appear to be exceptions for these requirements in at least some cells.

#### 1.1.1 MVB biogenesis

Exosomes demonstrate enrichment of the sphingolipids sphingomyelin and hexosylceramide relative to the lipid content of other cellular membranes, and the ceramide content of the outer endosome membrane appears to play an important role in exosome biogenesis. Neutral sphingomyelinase activity can hydrolyze sphingomyelin to ceramide to promote the inward curvature of the outer leaflet of the endosome membrane into the intraluminal space of the endosome to initiate or promote ILV formation.^13^ The role of ceramide in membrane invagination is supported by studies reporting that cells treated with either neutral sphingomyelinase or ceramide exhibit significantly increased rates of vesicle formation.^14^, ^15^ Neutral sphingomyelinase activity can be regulated by features commonly found in many tumor microenvironments, including hypoxia, oxidative stress, and elevated levels of proinflammatory cytokines (tumor necrosis factor‐α [TNF‐α], interferonγ [IFNγ], and interleukin‐1β [IL‐1β]).^16^, ^17^, ^18^ These results suggest potential mechanisms through by which changes in the tumor microenvironment can alter sphingolipid metabolism to promote exosome biogenesis, No studies, however, appear to have been conducted to address whether the attenuation of any of these factors inhibits exosome secretion through neutral sphingomyelinase‐ or ceramide‐dependent mechanisms. Inhibition or deletion of neutral sphingomyelinase activity does not attenuate MVB biogenesis in all cells, as demonstrated by a study in human melanoma cells,^19^ indicating that other mechanisms can compensate for its role in exosome biogenesis. It is not clear if these cells represent a special case since they also utilize ILVs to sort, process and assemble a major component of the melanocyte, a lysosome‐related organelle, during melanosome maturation, and employ an ESCRT‐independent, CD63‐regulated mechanism to conduct this process.^19^ However, a HRS‐ and CD63‐dependent mechanism appear to function in the HeLa cells,^20^ suggesting that similar mechanisms may be active in other cell types.

Multiple studies have reported alterations in sphingolipid metabolism in different cancer types, but it is not clear there is a consistent shift toward increased ceramide accumulation in these cancers or that the observed changes promote phenotypes that would be expected to enhance exosome biogenesis.^21^ This is particularly problematic since ceramide has multiple reported effects to signaling responses, including inflammation, drug resistance, and cell proliferation and death, where differences in ceramide fatty acid chain length have been linked to different phenotypes.^22^

ILVs are known to form through both ESCRT‐dependent and ESCRT‐independent mechanisms (Figure 1), although most studies to date have focused on the ESCRT‐dependent process. ESCRT‐dependent ILV formation and involves the sequential interactions of four ESCRT complexes (ESCRT‐0, ESCRT‐I, ESCRT‐II, and ESCRT‐III), the vacuolar protein sorting (VPS) 4‐vesicle trafficking 1 complex, and several ESCRT‐associated proteins, including ALIX. The proteins that form all of these complexes reveal strong evolutionary conservation from archaebacteria to mammals.^23^ The ESCRT machinery and ESCRT‐associated proteins perform three major functions: recognizing and binding material to be incorporated into ILVs, deforming the endosomal membrane and sorting selective cargoes into the forming vesicle, and regulating the closure and release of these invaginations to produce ILVs.

The ESCRT‐0 complex initiates the first step of the ILV biogenesis process by binding and promoting the aggregation of ubiquitinated proteins and regulating the localization of these proteins, and other ESCRT complexes, to clathrin‐coated microdomains on the endosome membrane. The ESCRT‐0 complex contains two subunits, HRS and STAM, both of which contain ubiquitin‐ and clathrin‐binding domains, while HRS also contains a Fab 1, YOTB, Vac 1, and EEA1 (FYVE) zinc finger domain that binds to phosphatidylinositol 3‐phosphate to recruit the ESCRT‐0 complex to the endosome membrane.^24^ ESCRT‐I and ESCRT‐II accumulation on the endosome membrane is regulated by sequential interaction of the ESCRT‐I protein VPS23/TGS101 with the ESCRT‐0 protein HRS and the ESCRT‐II protein VPS36/EAP45, so that depletion of HRS reduces endosomal accumulation of ESCRT‐I.^25^, ^26^ ESCRT‐I/ESCRT‐II promotes the involution of the endosome membrane and the sorting of cargo into the budding membrane space. ESCRT‐III, which further promotes endosome membrane deformation, is reported to assemble around VPS20/CHMP6, which interacts with the VPS25/EAP20 subunit of ESCRT‐II and with the abundant ESCRT‐III subunit SNF7/CHMP4 to initiate its assembly into oligomers that associate with other ESCRT‐III subunits. The assembled ESCRT‐III complex then associates with VPS4, which initiates scission of the budding membrane connection to release a mature ILV into the intraluminal endosome space.^27^, ^28^ However, one study has now reported that MVBs still form in cells where key subunits of all four ESCRT complexes have been depleted to provide maximal attenuation of ESCRT‐mediated exosome biogenesis,^29^ indicating that another mechanism can compensate for the attenuation of ESCRT‐mediated ILV biogenesis. Evidence now indicates that CD63 localized on endosome membranes can regulate ESCRT‐independent MVB biogenesis.^19^

It is not clear if cancer cell changes or tumor microenvironment stimuli can directly enhance the expression or activity of ESCRT‐dependent or ‐independent MVB biogenesis mechanisms, but the ESCRT‐0 protein HRS and the ESCRT‐I have been reported to demonstrate enhanced expressed in some tumor tissues, suggesting that such mechanisms may exist.^30^, ^31^, ^32^

#### 1.1.2 MVB intracellular transport

Mature MVBs can experience different fates depending on cellular conditions at the time of their formation, either trafficking to the cell periphery to permit a subsequent regulated fusion event with the plasma membrane that releases their cargoes of exosomes into the extracellular space or migrating to and fusing with lysosomes or autophagosomes to allow the degradation of their contents. The conditions, processes and factors that control the targeting of MVBs to each of these fates is not completely understood, although one group has now reported that interferon‐stimulated gene 15 (ISG 15)‐mediated ISGylation appears to play a key role in targeting mature MVBs to lysosomes or autophagosomes.^33^ In this study, VPS23/tumor susceptibility gene 101 (TGS101) ISGylation was found to be sufficient to promote MVB degradation without altering ILV biogenesis, although the authors acknowledged that several proteins found in exosomes and MVBs are also potential ISGylation targets, including several ESCRT‐III complex subunits, and could thus contribute to an ISGylation‐mediated MVB degradation mechanism. The expression of ISG15, and several other factors that regulate ISGylation, frequently exhibit dysregulation in different cancer types, but the mechanisms responsible for these effects and their outcomes are not clear, and may depend upon the cancer type.^34^

Endosomes and mature MVBs are transported along microtubules by dynein/dynactin or kinesin motor complexes in a process regulated the activity of RAB family GTPases, with the inward movement of maturing endosomes regulated by the action of RAB5 and outward transport of mature MVBs regulated by RAB7A.^35^ Evidence also suggests that RAB5 is involved in the regional enrichment of mitogenic receptors and exclusion of nutrient receptors, such as transferrin, which is removed from the maturing endosomes during dynein‐mediated inward migration.^36^ The microtubule‐binding protein Hook has also been implicated as a negative regulator of MVB development, due to its activity to prevent endosome maturation upon overexpression, and as a negative of MVB degradation, due to its activity to anchor MVBs to microtubules and inhibit their fusion with autophagosomes until RAB11 activity attenuates this interaction.^37^ Regulatory mechanisms responsible for MVB docking at the plasma membrane, or specific membrane domains, may be cell‐type specific or altered in cancer cells. For example, while RAB27 activity regulates MVB docking in multiple cancer cell lines, RAB35 regulates MVB docking in oligodendroglia cells, and epithelial cells can secrete exosomes containing different cargoes from their apical and basolateral membranes, which should necessitate the use of distinct MVB sorting and docking mechanisms at these surfaces.^38^, ^39^, ^40^, ^41^ There are more than 60 human RAB GTPases that associate with distinct intercellular membranes or membrane domains, and the activity of these proteins can be negatively and positively regulated by the activity of specific and potentially overlapping families of GTPase‐activating proteins and guanine nucleotide exchange factors,^42^ complicating the discovery of specific regulatory mechanisms acting on MVB trafficking. At least eight RAB GTPases have been implicated in the MVB secretion, although further studies are required to define the specific role(s) of these proteins in this process.^38^, ^39^, ^43^, ^44^ It is also not clear if the mechanisms that control MVB migration and docking undergo altered regulation as cells adopt a malignant phenotype, or whether phenotypes associated with altered expression of these factors are due primarily to changes in phenotypes directly regulated by exosome or as a result of other RAB‐mediated processes. RAB7A expression is reported to both promote and inhibit tumor development and progression, with different effects observed in different cancer types,^45^, ^46^, ^47^, ^48^ while RAB27 is reported to promote growth, invasion, metastasis, and chemoresistance in a variety of cancer types.^49^

#### 1.1.3 Exosome secretion

Exosome release from MVBs docked at the plasma membrane appears to be regulated by GTPase‐mediated interactions between soluble N‐ethylmaleimide‐sensitive factor attachment protein receptors (SNAREs) proteins present on the MVB and plasma membranes. Multiple SNARE proteins have been implicated in this process. For example, Ca^2+^ regulated MVB fusion with the plasma membrane is reported to involve interaction between plasma membrane‐associated SNAP‐23 and MVB‐associated vesicle associated membrane protein (VAMP7), VAMP8, or Syntexin‐4, depending upon the cell type.^50^, ^51^, ^52^ SNARE complex‐mediated membrane fusion events are a closely regulated process, and are partly controlled by SNARE phosphorylation events, which can alter SNARE protein complex interactions.^53^, ^54^

### 1.2 Regulation of exosome production by the tumor microenvironment

Most tumors demonstrate an enhanced degree of exosome secretion and several characteristics commonly associated with tumor microenvironments can regulatory exosome secretion rates. For example, most cancers demonstrate a characteristic metabolic shift toward aerobic glycolysis so that pyruvate produced during the first stage of aerobic glucose metabolism is converted to lactate, rather than entering the tricarboxylic acid cycle and undergoing complete oxidative phosphorylation.^55^ This diversion increases the amount of intracellular lactate available for synthesis of amino acids and lipids required for cell proliferation, but also necessitates an increase in glucose uptake to offset energy losses from partial glucose metabolism and ultimately an increase in lactate secretion to maintain cellular homeostasis.^55^ The tumor microenvironment thus tends to become nutrient poor, hypoxic and acidic as the metabolic demands of the tumor exceed the ability of tissue perfusion to maintain local homeostasis. Notably, multiple physiologic changes associated with the tumor microenvironment can induce cell stress pathways and increase exosome secretion rates, including hypoxia,^56^ extracellular acidification,^57^ and several proinflammatory mechanisms.^58^ Many of these processes are also known to interact, and to increase tumor resistance to cancer therapeutics.

#### 1.2.1 Hypoxia

Hypoxia can increase EV secretion rates and while the mechanism responsible is for such increases is not well understood, this effect appears to be hypoxia‐inducible factor 1α (HIF‐1α)‐dependent, since cells treated with HIF‐1α short hairpin RNA or specific‐inhibitors or with HIF‐1α deletions exhibit attenuated EV secretion when incubated under hypoxic conditions.^59^

#### 1.2.2 Acidification of the tumor microenvironment

Local acidification is a common feature of most tumors since acidic metabolites can accumulate due both to altered tumor metabolism and a relative reduction in the perfusion rate of the tumor. Lactic acid accumulation appears to be a significant contributor to this process, but other mechanisms have also been reported to influence tumor pH, including increased CO_2_ production by the pentose phosphate pathway.^60^

Several lines of evidence indicate that tumor acidification may play important roles in regulating the secretion and function of TDEs. For example, exposure to low pH is reported to stimulate exosome release and uptake through a proposed pH‐dependent effect on exosomal lipid composition,^61^ while inhibition of extracellular acidification by treatment with proton pump inhibitors is reported to attenuate TDE release.^62^ Local acidification may also directly increase TDE stability to promote exosome activity, since in vitro exposure of isolated exosomes to acidic pH has been reported to increase exosome RNA and protein recovery.^63^

#### 1.2.3 Growth factors

Dysregulated growth factor signaling, due to increased expression or altered receptor signaling pathway responses, is associated with enhanced tumor development and progression. Multiple growth factor signaling pathways now know to contribute to distinct tumorigenic responses in different cancer types. However, tumor‐associated epidermal growth factor receptor (EGFR) signaling is particularly notable in that enhanced expression of EGFR or its ligands is detected in most human carcinomas, EGFR expression is induced by local tissue hypoxia, and stimulation of EGFR signaling is reported to enhance exosome secretion.^64^, ^65^, ^66^

#### 1.2.4 Inflammation

Chronic inflammation is reported to regulate all stages of tumor development, from tumor initiation to metastasis,^67^, ^68^, ^69^ and a substantial body of research describes how tumor‐derived factors and the tumor microenvironment can regulate antitumor immune responses to favor tumor development.^70^ Chronic inflammation has also been linked to changes linked to increased risk of carcinogenesis, including telomere shortening, enhanced oxidative stress, and the attenuation of DNA damage repair systems.^71^

Multiple studies have examined TDE effects on tumor development and tumor microenvironment inflammation, as described in the following sections, but relatively few have examined how stress and proinflammatory tumor cell responses influence exosome secretion. However, recent work suggests that increased NLR Family Pyrin Domain Containing 3 (NLRP3) inflammasome activity, which plays a key role in innate immunity, can regulate EV secretion, with several reports demonstrating direct associations between NLRP3 inflammasome activation and enhanced exosome release rates and changes in exosome cargo compositions.^72^ NLRP3 inflammasome polymorphisms are also associated with the development of certain cancers, including breast cancer, melanoma, and hepatocellular carcinoma (HCC),^73^ and NLRP3 inflammasome inhibitors have been patented for potential cancer therapeutic applications.^74^

The NLRP3 inflammasome is a multi‐subunit cytosolic protein complexes that assembles upon exposure of its pattern recognition receptor, NLRP3, to a broad range of damage‐associated molecular patterns or responses induced by them, including extracellular adenosine triphosphate (ATP), crystalline complexes and protein aggregates, mitochondrial dysfunction and lysosomal damage.^75^ Assembly of the NLRP3 inflammasome complex forms a scaffold that induces the activation of procapase‐1 to catalyze the maturation of IL‐1β and IL‐18, which promote recruitment of immune cells and enhance the activity of natural killer (NK) and T‐cells, to stimulate a localized proinflammatory environment. Notably, caspase‐1 activation can also regulate the release of other leaderless proteins, including several known EV secreted proteins,^76^ implying that NLRP3 activation may also influence the composition of EV cargoes in affected cells.

NLRP3 activation by a signaling pathway that senses extracellular ATP or exposure to crystalline activators that mediate lysosome damage strongly induces the section of EV proteins,^77^, ^78^ while cells treated with the caspase inhibitor peptide pan‐caspase inhibitor Z‐VAD‐fluoromethyl ketone (ZVAD) exhibit reduced exosome secretion rates.^79^ The mechanism responsible for enhanced exosome secretion by NLRP3 activation is not clear, but multiple stimuli that promote NRLP activation induce calcium ion flux,^80^ which is known to induce EV secretion by promoting MVB maturation,^81^ suggesting that a common mechanism could stimulate both pathways to increase the export of proinflammatory and caspase‐1‐regulated factors. EVs secreted during inflammasome activation could also function to export other proinflammatory factors and, potentially, inflammasome components to reduce cell stress and the potential for such cells to undergo pyroptotic cell death, while the exosomes secreted by these cells could increase the proinflammatory phenotype of recipient cells in the local microenvironment.^82^ Several mechanisms may regulate increased NLRP3 activation in cancer cells to promote EV secretion, including mitochondrial dysfunction, endoplasmic reticulum (ER) stress, and factors released by these organelles.

Mitochondrial dysfunction, characterized by dysregulated oxidative metabolism, is frequently associated with increased reactive oxygen species (ROS) production. ROS produced during oxidative phosphorylation represent a major endogenous source of genetic damage and mitochondrial mutations are linked to mitochondrial dysfunction, increased ROS production, and enhanced tumor proliferation and metastasis.^83^, ^84^ Conversely, cell stresses associated with most cancers, such as hypoxia and elevated metabolic rates, are known to increase mitochondrial ROS production.^85^ Mitochondrial dysfunction promotes three processes that can induce NLRP3 inflammasome complex assembly: enhanced ROS production, translocation of the phospholipid cardiolipin to the outer mitochondrial membrane, and cytosolic release of oxidized mitochondrial DNA.^86^ NLRP3 has also been shown to colocalize with the ER in resting cells, but to migrate to junctions between mitochondria and ER upon inflammasome stimulation,^87^ which should enhance the ability of NLRP3 to detect ROS released by damaged and dysfunctional mitochondria.

A disturbance in ER homeostasis can promote in ER stress, which is characterized by disruption of normal protein folding events, decreased ER secretory function, and the accumulation of misfolded and unfolded proteins in the ER lumen, and results in the induction of the unfolded protein response. Notably, low nutrient and acidic pH conditions frequently found in tumor microenvironments can inhibit normal N‐linked glycosylation and side chain interactions to alter protein folding and activate the unfolded protein response.^88^ This can, in turn, inhibit genomic DNA repair mechanisms to increase the affected cell's susceptibility to DNA damage.^89^ ER stress is also reported to induce mitochondrial dysfunction through an NLRP3‐mediated mechanism.^90^

Mounting evidence suggests that inflammasome activity can regulate exosome secretion and composition, and that inflammasome activation can be induced by multiple stimuli associated with tumor development and exosome secretion. However, while these findings are suggestive, definitive studies have not been performed to address the relative contribution(s) of inflammasome activation in the regulation of TDE biogenesis and secretion.

### 1.3 Exosome cargo sorting

#### 1.3.1 Protein sorting mechanisms

Multiple studies now demonstrate that selective sorting processes regulate the accumulation of specific membrane and cytosolic proteins into developing ILVs during MVB biogenesis. Several reports indicate that the ubiquitination state of a protein, which is known to target both cytosolic and membrane proteins to early endosomes for subsequent degradation, is also an important determinant of selective protein accumulation into ILVs during MVB biogenesis. Further, it appears MVBs or a subset of ILVs within an MVB can be shunted to different pathways via a regulatory mechanism that are still not fully clear to target them to secretory or degradative fates.

Mounting evidence also indicates that ESCRT complex proteins play an important role in sorting ubiquitinated proteins into developing ILVs. For example, the ESCRT‐0 protein HRS, which plays a key role in the initiation of ILV development, contains a ubiquitin interaction motif that promotes the accumulation of ubiquitinated proteins at clathrin‐coated membrane domains of early endosomes, and that mutations that block this ubiquitin‐binding activity also abolish the sorting function of HRS.^91^, ^92^ The ESCRT‐0 protein STAM is also reported to demonstrate ubiquitin‐binding activity, as are components of the ESCRT‐I and ESCRT‐II complexes.^93^ The tetraspanin protein CD63, which is expressed on endosome membranes and most exosomes, is also reported to regulate the selective sorting of ILV cargoes via an ubiquitin‐independent process.^19^

#### 1.3.2 RNA sorting mechanisms

Exosomes carry cargoes that contain multiple distinct RNA species and are primarily enriched in small noncoding RNAs, including miRNAs, and such repertoires can markedly differ between exosomes isolated from normal and cancer cells of the same lineage.^94^, ^95^ Multiple RNA binding proteins have been implicated in selective transport of miRNAs species, including SYNCRIP and hnRNPA2B1.^96^, ^97^ However, it is not clear if there is selective enrichment of specific RNA transcripts by RNA sorting mechanisms during exosome ILV loading. Notably, though, several groups have reported that miRNAs with tumor suppressor activity are enriched in TDEs and that suppression of exosome release increases the intracellular concentration of these miRNAs while reducing the malignant phenotype of the affected tumor cells. Exosome secretion may thus play an important role in tumorigenesis by reducing the effective expression of tumor suppressor miRNAs.

#### 1.3.3 DNA sorting mechanisms

Cancer‐associated mutant alleles represent obvious DNA biomarker targets for analyses of exosome cargoes, and in addition to their potential value as diagnostic and prognostic markers, may also provide critical information to inform treatment decisions. However, other exosome DNA parameters may also be informative. Exosomes derived from multiple different cancer types demonstrate a marked increase in overall DNA content,^98^ although the mechanism(s) responsible for this change and its functional impact(s) of are not fully understood. Both the genomic and mitochondrial DNA content of exosomes are reported to increase in exosomes secreted from cancer cells. Blockade of exosome biogenesis or secretion in senescent primary human fibroblasts was found to cause cytoplasmic accumulation of nuclear DNA fragments and provoke a ROS‐dependent DNA damage response regulated by activation of the stimulator of interferon genes pathway, which resulted in irreversible cell‐cycle arrest and apoptosis.^99^ DNA analysis of these exosomes identified DNA fragments derived from all chromosomes but did not detect a mitochondrial DNA contribution. Nuclear envelope instability, particularly in micronuclei arising from chromosome breakage or mitotic errors, represents a likely candidate mechanism for the increase in cytosolic release of nuclear DNA in senescent or cancer cells,^99^ but the mechanism(s) responsible for packaging this material into exosomes is not clear, nor whether there is differential regulation of this process upon cell senescence or cancer development and progression. While this study did not detect mitochondrial DNA in during its analysis of exosome DNA content from senescent cells, another study found that tumor‐associated exosomes can package significant amounts of mitochondrial DNA, which could rescue recipient cancer cells from metabolic dormancy by stimulating oxidative phosphorylation.^100^ Notably, this study also found that exosomes isolated from cancer‐associated fibroblasts preferentially transferred mitochondrial DNA to cancer stem cells (CSCs) relative to non‐CSC‐like populations to increase their potential for self‐renewal. The mechanism responsible for the enhanced packaging of cellular DNA into secreted exosomes is not clear, although it does not appear that the mechanism responsible targets specific loci since exosome‐associated DNA fragments demonstrate a random distribution across the entire genome.

### 1.4 Therapeutic targeting of exosome secretion for tumor control

Many of the factors involved in exosome biogenesis and secretion demonstrate a strong degree of evolutionary conservation.^23^ It thus appears unlikely that cellular mechanisms that regulate exosome secretion in malignant cells markedly differ from those employed by nonmalignant cells. Since protein components of the exosome secretory pathway are conserved in single cell organisms, it appears likely that it may have originally functioned as a means to dispose of excess or potentially toxic cellular material. In multicellular organisms, however, intracellular communication events mediated by exosomes regulate the local microenvironment, and distant tissue niches and influence immune function, as discussed later in this review.

Enhanced exosome secretion rates observed from tumor cells appear to be induced in response to changes in cell phenotypes (eg, elevated cytosolic DNA) and environmental conditions (eg, tissue hypoxia) that can also affect nonmalignant cells and tissues. It is less clear what specific processes regulate observed differences in the content of exosomes derived from malignant vs nonmalignant cells. Some of the enrichment observed in TDEs may reflect increased cellular abundance (eg, cytosolic DNA)^99^; other factors may be enriched due to a joint increase in their expression and the expression of their packaging factors (eg, miRNAs)^94^, ^95^, ^96^, ^97^; while the enrichment of others may be solely regulated by the overexpression of their specific packaging factors. Therapeutic approaches that target general exosome secretion pathways may not be feasible since most cells secrete exosomes and systemic suppression of exosome secretion may thus give rise to significant side effects. Better understanding of processes involved in packaging certain factors could uncover therapeutic targets that may give rise to fewer or less severe side effects. The following sections describe specific TDE‐regulated mechanisms that may offer better opportunities for therapeutic intervention.

## 2 TDE‐INDUCED IMMUNE RESPONSES

Recognition of tumor antigens by the immune system is essential for effective antitumor responses, and T‐cells reactive against specific tumor antigen‐derived epitopes are present both in the circulation and at tumor sites.^101^ The ability of tumor epitopes to trigger successful cytotoxic T‐lymphocyte (CTL) responses in both in vitro studies and animal models has led to the rapid and successful translation of this information to clinical trials for various malignant diseases.^102^, ^103^ However, the ability of immune responses generated against such antigens to arrest tumor progression and prevent tumor recurrence can be restricted in some cancer patients.^104^ The underlying mechanisms for this discrepancy may vary depending on the nature of the tumor and the tumor microenvironment, and be regulated by editing of tumor antigens displayed in the initial and later stages of tumor development by the immune system.^105^ Effective treatments that can extend the utility of tumor antigens in cancer therapy are urgently needed to improve patient outcomes. TDEs are of interest due to recent research that implicates them in multiples mechanisms that can directly regulate antitumor or immune suppressive responses.

**Figure 2:**
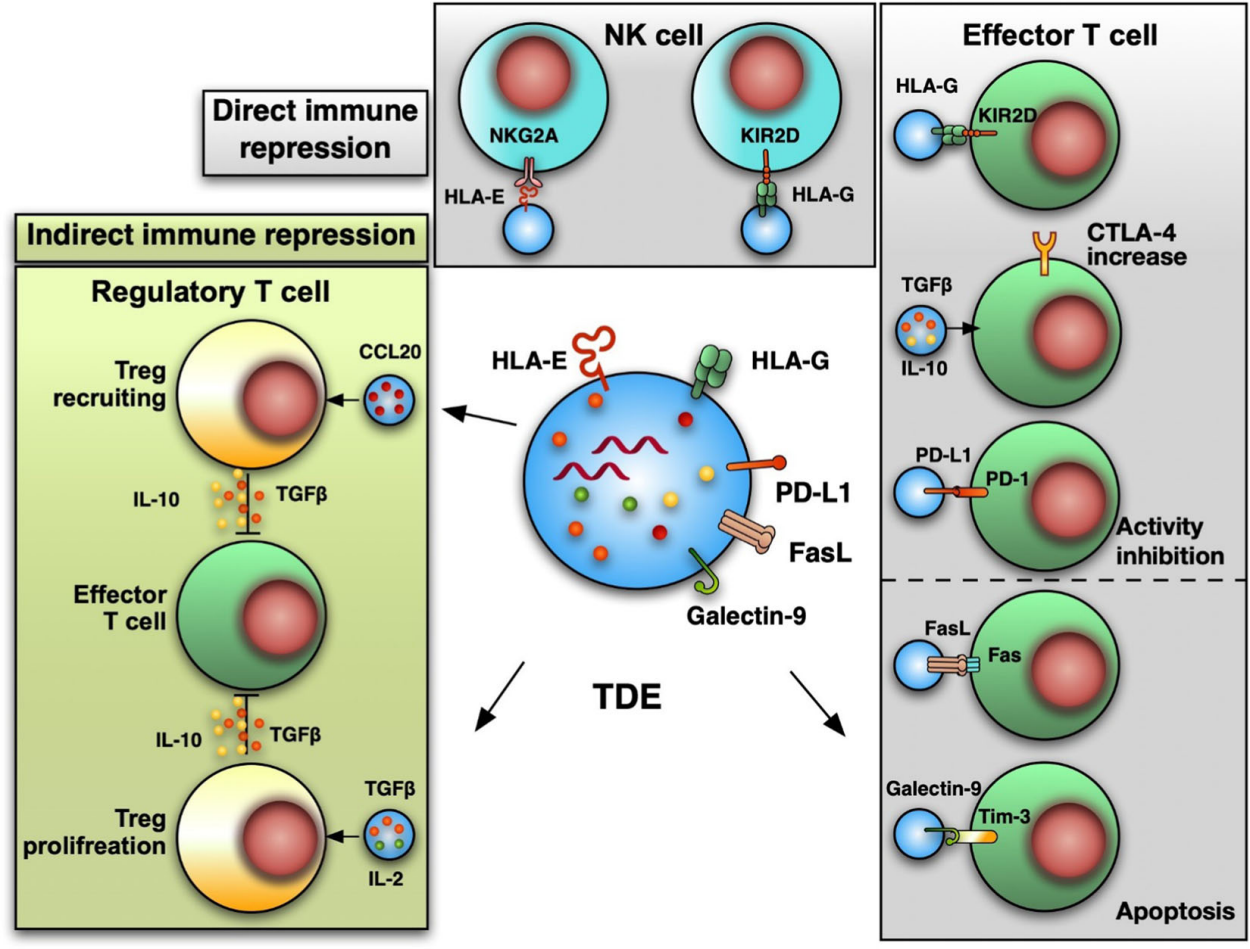
Tumor‐derived exosome (TDE)‐mediated mechanisms to suppress immune function. For natural killer (NK) cells, TDE factors (HLA‐G and HLA‐E) can suppress cell functions by interacting with surface receptors (NK cell inhibitory receptor 2A [NKG2A] and killer cell immunoglobulin‐like receptor 2D [KIR2D]). For effector T‐cells, TDEs can suppress cell function though HLA‐G:KIR2D or programmed cell death ligand 1:programmed death‐1 (PD‐L1:PD‐1) interactions or by releasing interleukin 10 (IL‐10) or transforming growth factor β (TGF‐β) or promote apoptosis through FasL:Fas or Galectin‐9:Tim3 interactions. TDEs can also indirectly suppress effector T‐cells releasing C‐C motif ligand 20 (CCL20) to promote Treg recruitment or TGF‐β and IL‐2 promote regulatory T‐cell (Treg) proliferation [Color figure can be viewed at wileyonlinelibrary.com]

Multiple immune cells are sensitive to TDEs‐mediated regulatory effects,^106^ but positive and negative TDE effects on immune cell functions predominantly act through different cell types, as discussed in later sections. TDEs containing antigens or antigen‐MHC complexes can directly transfer these factors to dendritic cells (DCs) or to other antigen presenting cells. This transfer can enhance the ability of these cells to present TDE‐derived tumor antigens on their MHC‐I/CD8 or MHC‐II/CD4 complexes to regulate tumor‐directed T‐cell responses.^107^, ^108^, ^109^, ^110^, ^111^, ^112^, ^113^ Conversely, TDEs can directly inhibit effector T cells by presenting inhibitory molecules (eg, PD‐L1 or FasL) that inhibit T‐cell activation or proliferation, or promote T‐cell apoptosis (Figure 2).^114^, ^115^, ^116^, ^117^ Exposure to TDE‐derived factors (eg, transforming growth factor β [TGF‐β]) in TDEs can also inhibit effector T‐cell function,^118^, ^119^ and stimulate regulatory T‐cells (Tregs) to proliferate in tumor microenvironment to inhibit the antitumor activities of effector T cells (Figure 2).^120^ These potentially conflicting TDE factors and effects (Table 1) appear to ultimately favor immune suppression since TDEs also exhibit activity to suppress the maturation and activity of DCs and can attenuate T‐cell and NK cell activation.

**Table 1:** Summary of TDE‐associated factors with reported immune cell effects

Function	Target cells	Source cells	Factor	Function	Refrences
Immune activation	DC	Prostate cancer	EGFR		107
	DC	Breast cancer	HER2		108
	DC	Colon cancer	CEA	TAA complex	109
	DC	Mesothelioma	Mart1		110
	DC	Multiple cancers	MHC I/II		111
	DC	Melanoma	CD80/86	Costimulatory	112, 113
	T cell; NK cells	B‐lymphoma, multiple cancers	HSP	Heat shock protein	121
Immune repression	NK cells	Renal CSCs, melanoma	HLA‐G, HLA‐E	Nonclassical MHC‐I	122, 123
	CD8 T cell	Melanoma, prostate cancer	FasL	Death receptor ligand	116, 117
	CD8 T cell	Multiple cancers	PD‐L1	PD‐1 ligand	114, 115, 124
	CD8 T cell	Leukemia	TRAIL	tumor necrosis ligand	125
	CD8 T cell	Fibroblast	TGFβ	Cytokine	118, 119
	Treg	Nasopharyngeal carcinoma	CCL20	Cytokine	120
	CD8 T cell	HNSCC	IL‐10	Cytokine	118, 119
Cancer metastasis	Lung fibroblasts	DCIS	Integrin α6β4 Integrin α6β1	Integrin	126
	Kupffer cell	DCIS	Integrin αvβ5	Integrin	126
	Kupffer cell	PDAC	MIF	Cytokine	127

Abbreviations: BLBC, basal‐like breast cancer; CCL20: C‐C motif ligand 20; CEA, carcinoembryonic antigen; DC, dendritic cell; DCIS, ductal carcinoma in situ; EGFR, epidermal growth factor receptor; FasL, Fas ligand; HCC, hepatocellular carcinoma; HER‐2, human epidermal growth factor receptor 2; HNSCC: head and neck squamous cell carcinoma; HSP, heat shock protein; MHC, major histocompatibility complex; MIF, macrophage migration inhibitory factor; TRAIL, tumor necrosis factor‐related apoptosis‐inducing ligand; Treg, regulatory T cell; PDAC: pancreatic ductal adenocarcinoma; PD‐1, programmed death‐1; PD‐L1, programmed cell death ligand 1; TAAs, tumor‐associated antigens; TGF‐β, transforming growth factor β.

### 2.1 TDE‐associated antigens

TDEs carry factors that can regulate innate and adaptive immune responses, and tumor‐associated antigens (TAAs) and immune‐regulatory molecules can be selectively packaged into TDEs by their parental tumor cells. Examples of exosome‐enrichment of TAAs include EGFR,^107^ human epidermal growth factor receptor 2 (HER‐2),^108^ carcinoembryonic antigen (CEA),^109^ and Mart‐1/MelanA.^110^ TDEs can also express multiple proteins related to antigen‐presentation and adaptive immune responses. These include class I and II major histocompatibility complex (MHCI and MHCII) proteins and costimulatory proteins (CD80/86); death receptor ligands, such as Fas ligand (FasL) and tumor necrosis‐factor‐related apoptosis‐inducing ligand (TRAIL); and other regulatory factors, such as prostaglandin E2 (PGE2).^116^

### 2.2 TDE‐induced T‐cell responses

Several studies have shown that TDEs enriched in MHC‐I proteins or heat shock proteins (HSPs) represent a novel pathway through which tumor antigens can be transferred to DCs to prime T‐cell activation responses. When TDEs are loaded onto human DC cells, TDEs bearing MHC‐I proteins and the lysosome‐associated membrane protein‐1 were found to transfer tumor antigens, and to induce antigen‐specific CTL activation responses in vitro and induce CD8^+^ T cell‐dependent antitumor effects in a mouse tumor model.^110^ In the same study, TDEs derived from different cancer cell lines were found to induce cross‐protective immune responses, indicating that TDEs may contain shared tumor‐rejection antigens that can induce antigen‐specific immune responses independent of the tumor MHC type match. This cross‐presentation ability was also demonstrated in a murine HCC model and in vitro cultures using human cells.^104^ Given the inherent tolerogenic character of the human liver, patients with HCC are poorly immunogenic and have low response rates to DC‐based immunotherapy.^111^ However, HCC TDEs were found to elicit DC‐mediated antigen‐specific responses from CD8^+^ T‐cells and CD4^+^ T‐cells independent of MHC type, as demonstrated by cross‐protective reactivity against various mouse and human HCC cells.^104^ HCC‐derived TDEs were also found to promote effective responses against pancreatic cancer cells. TDEs from ascites fluid of melanoma patients delivered Mart1 tumor antigens to DC cells to induce CTL responses for Mart1‐specific clones.^128^ The consensus from these reports indicates that TDEs can induce effective antitumor immune responses, and that these responses are independent of the MHC type match.

Recent studies have compared the mode of tumor antigen capture by DC cells and whether the same antigen could induce variable antitumor effects when encountered through different delivery mechanisms. One study found that when cancer cells were engineered to express the same antigen on their cell surface, on their exosomes, or in a secreted form, the cells expressing the antigen on their exosomes induced a stronger antigen‐specific in vivo immune response.^129^ These TDEs were found to induce multiple antitumor immune responses, including antigen‐specific CD8^+^ T‐cell responses, CD4^+^ T‐cell help, antigen‐specific antibodies, and a decrease in the tumor abundance of Tregs. These results imply that DC cells can capture and present tumor antigens more efficiently in vivo when they are presented in TDEs. However, the ability of TDEs to modulate antitumor immune responses may also depend upon TDE abundance, since glioma TDEs incubated with healthy donor peripheral blood mononuclear (PBMCs) can produce distinct immune effects depending upon the TDE concentration.^130^ A low TDE concentration was found to induce an activated DC phenotype that elicited T‐cell proliferation, differentiation, and migration, while a higher TDE concentration stimulated an immunosuppressive phenotype.^130^ Further investigation is therefore required to better understand how TDEs and TDE‐derived antigens interact with the immune system to regulate antitumor responses.

HSPs are overexpressed in a variety of human cancers where they are associated with tumor development and metastasis, and are a common component TDE cargoes. TDEs derived from heat‐shocked mouse B‐lymphoma cells^121^ and heat‐stressed colon carcinoma cells^109^ are enriched in HSP and have increased antitumor effects compared TDEs from untreated cells, both in vitro and in vivo. Strong proinflammatory helper T‐cell (Th1) immune responses may be particularly relevant for HSP‐70‐enriched TDEs extracted from heat‐treated mouse colon carcinoma cells, which enhance cancer cell elimination in both autologous and allogeneic hosts in conjunction with a decrease in the Treg population.^131^ HSP‐70^+^ TDEs were also observed to stimulate NK cell migratory and cytolytic activity.^132^ It has also been reported that in vitro treatment of human HCC cell with anticancer drugs efficiently upregulates the expression of HSPs on their TDEs, and that these TDEs efficiently stimulate NK cell‐mediated cytotoxicity.^133^ HSPs can tightly bind antigenic peptides and facilitate their transfer to DC cells,^134^ and HSP‐enriched TDEs may thus promote antigen‐specific antitumor T‐cell responses by enhancing the transfer of cancer antigens to DCs. Although the underlying mechanisms responsible for HSP release remain to be elucidated, heat‐stressed TDEs enriched in HSPs may be an efficient means to induce immune responses in vaccines intended for tumor immunotherapy.^135^ TDEs are a source of multiple tumor antigens and can be engineered to redirect their effects on immune cells to elicit or augment DC‐mediated immunotherapy approaches designed to promote CTL and NK cell cytolytic immune responses. Exosomes bearing tumor antigens are currently being investigated as novel cell‐free immunotherapeutic tumor vaccines in a number of clinical trials.^136^

## 3 TDE‐MEDIATED IMMUNE SUPPRESSION

TDEs have been reported to exhibit both negative and positive effects on immune responses, but the in vivo interactions and ultimate balance between these effects is not clear, and may depend upon the cells and tumor microenvironment from which a TDE population originates. Nevertheless, it seems clear that TDEs are capable of mediating multiple mechanisms that can promote tumor growth and metastasis by negatively regulating immune responses that would otherwise limit these phenotypes (Figure 2).

### 3.1 TDE‐mediated suppression of effector T‐cell activity

The mechanisms that tumors employ to escape the negative regulatory effects of the host immune system have long been a subject of great interest.^137^ One important mechanism is suppression of T‐cell activity, either by the induction of effector T‐cell apoptosis or the inhibition of T‐cell proliferation.^138^ Several new cancer treatments aim to overcome tumor‐mediated T‐cell suppression by employing monoclonal antibodies (mAbs) to target key immune checkpoint proteins. Current targets include CTL‐associated protein 4 (CTLA‐4) and programmed death‐1 (PD‐1) or programmed cell death ligand 1 (PD‐L1). Blockade of these proteins can restore immune activity. For example, the interaction PD‐1 on a CD8^+^ T‐cell with its ligand PD‐L1 directly inhibits the effector functions of the CD8^+^ T‐cells by inducing regulatory pathways that control proliferation, survival, cytokine production, and cytotoxicity.^124^, ^139^, ^140^ Restoration of antitumor responses by blocking these immune checkpoint inhibitors, has been shown to improve outcomes in some cancer patients.

TDEs carry an array of tumor‐derived factors that can suppress T‐cell activity, and TDEs, but not exosomes from nonmalignant cells, can induce the apoptosis of activated CD8^+^ T‐cells (Figure 2). Exosome PD‐L1 expression, but not the soluble PD‐L1 level, is associated with disease progression in patients with head and neck squamous cell carcinomas (HNSCCs), and the circulating PD‐L1^+^ TDE level appears to be a useful metric of disease and immune activity in HNSCC patients.^114^ Metastatic melanomas release TDEs that express PD‐L1 on their surface. TDE‐associated PD‐L1 and circulating IFNγ levels positively correlate in patients with metastatic melanoma, and IFNγ simulation increases PD‐L1 expression on these TDEs to suppress the function of CD8^+^ T‐cells and facilitate tumor growth (Figure 2).^115^ TDE PD‐L1 levels also vary during the course of anti‐PD‐1 therapy and the magnitude of the TDE PD‐L1 increase during early treatment, was found to stratify clinical responders from nonresponders.^115^, ^124^

The ESCRT‐associated protein ALIX is reported to be a critical regulator of both EGFR activity and PD‐L1 surface presentation in basal‐like breast cancer cells. ALIX depletion was found to extend stimulation‐induced EGFR activity, resulting in defective MVB‐mediated PD‐L1 trafficking, reduced exosome‐mediated PD‐L1 secretion, and the redistribution of PD‐L1 to the cell surface.^124^, ^141^ Research is now being conducted to test the ability of TDE‐expressed immune checkpoint inhibitors to target and block these immunosuppressive mechanisms to enhance the ability of endogenous immune cells to deliver robust and effective clinical responses.

Detailed characterization of inhibitory immune checkpoint receptors and ligands expressed on cancer cells and cancer‐directed immune cells is necessary to assess the potential efficacy of specific immune checkpoint inhibitors. Better understanding of the immunosuppressive contributions arising from TDEs and exosomes derived from immune cells is critical to develop such strategies.^141^

### 3.2 TDE‐mediated upregulation of Treg activity

Tregs, characterized by expression of the transcription factor forkhead box protein 3 (FOXP3), play key roles in immune homeostasis and self‐tolerance,^142^ where they function to limit proinflammatory responses. These cells were first implicated in the regulation of autoimmune diseases but can also limit other immune responses, including cancer‐mediated CTL responses. Tregs can potentially suppress antitumor CTL responses to permit tumor growth, and increased Treg concentrations are detected in tumors or the circulation of cancer patients who exhibit poor prognosis.^143^ However, the cellular and molecular mechanisms that govern the induction, expansion, survival, and function of Tregs and their site of origin (thymic or peripheral) in malignant disease is not well understood.^142^

Emerging evidence indicates that TDEs can induce Treg responses to promote Treg expansion and enhance their suppressor functions.^118^, ^119^ Treatment of PBMCs from healthy donors with IL‐2 induces the expression of CD25, the high‐affinity IL‐2 receptor α chain, in all lymphocyte subsets to induce cell proliferation. However, this effect is selectively inhibited in the CD8^+^ T‐cell population when PBMCs are co‐incubated with TDEs, indicating that TDE exposure selectively impairs CTL effector cells (CD8^+^), without affecting the Treg population (CD4^+^). TDE exposure did not inhibit expression of the Treg marker FOXP3 and enhanced the ability of the exposed Tregs to respond to IL‐2 and inhibit T‐cell proliferation.^118^

Better understanding of the signaling pathways and factors that can converge to promote an immune response is an important challenge for developing effective immunotherapies. For example, both CTL and Treg responses are partially regulated by IL‐2 exposure, but Tregs respond to low IL‐2 concentrations that do not stimulate CTL responses, while CTL response tend to dominate at higher IL‐2 concentrations. Since TDE exposure can preferentially enhance Treg responses to IL‐2, administering IL‐2 to cancer patients with the goal of promoting CTL responses may promote a Treg response and the immune escape of the tumor rather than a tumor‐limiting CTL response.^144^ However, TDEs also carry other factors, including TGF‐β or IL‐10, that are reported to regulate Treg and effector T‐cell homeostasis (Figure 2).^118^, ^119^ TGFβ‐specific neutralizing Abs are reported to significantly inhibit the ability of TDEs to promote the expansion and function of Tregs. Expression of the chemokine C‐C motif chemokine ligand 20 on nasopharyngeal carcinoma‐derived TDEs is reported to recruit Tregs into the tumor microenvironment (Figure 2). These TDEs can also induce Treg expansion, and increase their suppressor functions.^120^

Circulating Treg concentrations are elevated in patients with head and neck cancer and ovarian cancer relative to healthy subjects, and serum from these patients are also highly enriched in TDEs.^119^ Studies have now demonstrated that TDEs can promote the in vitro expansion of Tregs from precursors (inducible costimulator and ICOS ligand differentiation model^145^) in the peripheral blood^146^ and the conversion of CD4^+^CD25^−^ T‐cells into CD4^+^CD25^high^FOXP3^+^ Treg.^119^ TDEs have also been reported to inhibit the proliferation of pre‐activated CD8^+^ T‐cells and to activated CD4^+^ T‐cells.^146^ TDE‐mediated immunosuppressive activity appears to be related to their effects to upregulate FasL, IL‐10, TGF‐β, CTLA‐4, granzyme B, and perforin expression by Tregs, which been found to be resistant to TDE‐induced apoptosis.^119^ While mounting evidence indicates that TDEs can regulate Treg and CTL functions, more studies are required to evaluate better define the key factors involved and potential pathways that mediate these functions.

### 3.3 TDE‐induced T‐cell apoptosis

Early studies indicated that coculture of activated T‐cells with tumor cells, their supernatants or serum samples from cancer patients, but not with normal fibroblasts or their supernatants, could induce T‐cell apoptosis.^117^, ^147^ Subsequent studies revealed that TDEs isolated from culture supernatants and sera could induce the apoptosis of activated CD8^+^ T‐cells. These observations were explained in various cancers by a mechanism in which the presence of MHC‐I and a membrane‐associated form of FasL on the TDE surface could suppress T‐cell responses by inducing apoptosis,^116^, ^117^ as indicated by the ability of anti‐Fas or anti‐MHC class I antibodies to block this effect.^116^, ^147^

In the circulation of cancer patients, the majority of lymphocytes are Fas^+^ T‐cells and it is clear that the Fas/FasL pathway is directly involved in spontaneous apoptosis of circulating Fas^+^ T‐cells.^148^ Apoptosis of tumor‐infiltrating lymphocytes (TILs) found in tumor sites is also related to FasL expression on the surface of tumor cells.^149^ FAS/FASL‐mediated apoptosis leads to a turnover of T‐lymphocytes in the circulation and at tumor sites and, subsequently, a functional lymphocyte imbalance and reduction of the immune competence in these patients. TDEs are likely to be at least partially responsible for spontaneous apoptosis of CD8^+^ T‐cells in vivo through their expression of FASL (Figure 2).^149^ The apoptotic effect of TDEs from oral and ovarian cancers has been attributed to caspase‐3 cleavage, cytochrome C release, loss of mitochondrial membrane potential and signaling defects in CD3‐ζ chain and JAK3 in T‐cells.^147^, ^150^ Observation of FasL^+^ TDEs in the circulation as well as at tumor sites indicate that the immunoinhibitory influence of tumors might extend far beyond the tumor sites and may be a systemic effect.

Early ex vivo studies revealed that T‐cells isolated from previously treated head and neck cancer patients with no evidence of current disease exhibited higher levels of spontaneous apoptosis than T‐cells from normal donors.^151^ The level of ex vivo apoptosis could not, however, distinguish patients in subgroups treated with surgery alone or with surgery plus chemotherapy and/or radiotherapy, or metastatic melanoma patients.^151^ The finding emphasizes the long‐lasting nature of cancer‐induced disturbances in T‐cell homeostasis, and suggests that aberrations in T‐cell homeostasis can occur or persist in the absence of overt tumor growth in patients with no evidence of disease following oncologic therapy. The basal level T‐cell apoptosis might thus be viewed as an important indicator for therapeutic interventions aimed at protecting of immune cells from apoptosis. The role of TDEs in this process is not known, but their known activity to enhance T‐cell apoptosis represents a potential mechanism that could explain this phenomenon. Circulating TDEs with “high FasL” expression were associated with advanced stage (T3/T4) tumors in head and neck cancer patients, while TDEs with “low FasL” levels were associated with early stage (T1/T2) tumors.^147^ TDEs from the serum of patients with active disease, primary HNSCC without prior treatment and recurrence HNSCC, also expressed higher FasL levels than those of patients with no evidence of disease or normal controls.^152^ These preliminary findings highlight the potential ability of TDEs to predict disease progression.

TDEs may regulate both CD4^+^ T‐cell and CD8^+^ T‐cell homeostasis. In addition to the reported effect of FasL^+^ MHC‐I^+^ TDEs to induce CD8^+^ T‐cell apoptosis, a more recent study has reported that TDEs that express MHC‐II and galectin‐9, a ligand of the death receptor Tim‐3, are detectable in the plasma of patients with nasopharyngeal carcinoma, and can induce apoptosis in mature Th1 lymphocytes (Figure 2).^153^ This effect was inhibited by both anti‐Tim‐3 and anti‐galectin‐9 antibodies. These observations imply that TDE‐mediated regulation of lymphocyte apoptosis at tumor sites and in the periphery can lead to rapid and selective tumor‐specific lymphocyte turnover, resulting in a loss of effector cells to contribute to the immune escape of tumors to allow their enhanced development and progression.

### 3.4 TDE‐induced natural killer cell suppression

Immunoregulatory mechanisms that lead to the immunoediting of T‐cells are complex. IL‐2 plays a dominant role in the homeostasis of the immune system and can promote the survival, proliferation, and functional differentiation of effector and suppressor cell populations, including CD8^+^ T‐cells, NK cells and CD4^+^ CD25^+^ Tregs.^144^, ^154^ Conversely, TGF‐β1 may be essential for T‐cell‐mediated immune suppression.^155^ One study has reported that TDEs derived from tissue or pleural fluid of patients with advanced malignant pleural mesothelioma could strongly inhibit IL‐2‐driven lymphocyte proliferation.^118^ This effect was seen in all cell subsets and was related to TGF‐β1 expression on TDEs. In another study of mesothelioma patients, downregulation of NK group 2D (NKG2D) activating receptor expression on CD8+ T‐cells and NK cells was found to partly rely on TDEs carrying MHC‐I‐related NKG2D and killer cell immunoglobulin‐like receptor 2D (KIR2D) ligands, such as the human leucocyte antigen (HLA)‐G class.^156^ HLA‐G is a nonclassical HLA class I molecule, and can potently suppress the effector functions of immune cells of the innate and adaptive immune system. HLA‐G interacts with its cognate inhibitory receptor NKG2D,^157^ and this interaction is considered to play a crucial role in the network of immune‐regulatory tolerance mechanisms.^158^ TDEs isolated from prostate, breast and B‐lymphoblastoid cell lines induce deficient NKG2D expression,^159^ while circulating TDEs derived from prostate cancer patients selectively induce downregulation of NKG2D on CD8^+^ T‐cells and NK cells.^160^ Thus, as observed in previous studies with bulk tumor cells,^161^ TDEs expressing NKG2D ligands bind to NKG2D receptors on CD8^+^ T‐cells and NK cells to contribute to the impaired cytotoxic effector functions of these cells (Figure 2). HLA‐G binding to KIR2D on NK also inhibits the cytolytic function of these cells (Figure 2).^162^ TDEs isolated from the plasma of patients with advanced melanoma skew CD14^+^ monocyte differentiation toward CD14^+^HLA‐DR^−/low^ cells that demonstrate TGF‐β‐mediated suppressor activity on T‐cell functions. In patients with EBV‐associated malignancies, LMP1‐containing TDEs may mediate immunosuppressive effects on TILs.^163^ HLA‐E is another nonclassical MHC‐I molecule, and can bind to the NK cell inhibitory receptor 2A (NKG2A)^164^ to inhibit T‐cell or NK cell‐mediated cytolytic activity^162^, ^165^, ^166^ and lead to cancer immune escape (Figure 2). HLA‐E is upregulated in breast cancer,^167^ melanoma,^168^ prostate cancer,^166^ and colon cancer.^169^ Our review of the literature did not reveal any reports of T‐cell or NK cell effects mediated by TDE‐associated HLA‐E. However, it appears reasonable to assume that both HLA‐G and HLA‐E are expressed on the surface of TDEs to produce similar regulatory effects, although studies need to be performed to address this hypothesis. These data highlight that the shedding of TDEs from tumor cells is an important cell‐to‐cell contact independent mechanism that enables the evasion of systemic immune responses, although it is still unclear which specific pathways are dominant in TDE‐mediated regulation.

### 3.5 TDEs promote the immune escape of tumor cells

**Figure 3:**
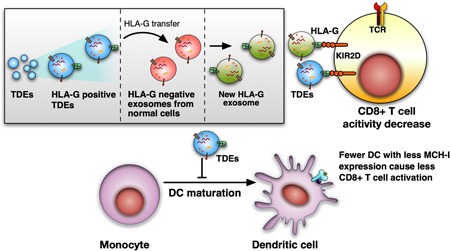
Tumor‐derived exosome (TDE)‐mediated effects on innate and adaptive immune cells. TDEs can transfer the human leucocyte antigen (HLA)‐G class to exosome from normal cells, and both can interact with effector T‐cells through HLA‐G:KIR2D interactions to suppress CD3 expression and CD3‐mediated T‐cell activation. TDEs call also suppress dendritic cell (DC) maturation, reducing the number of DCs and their expression of costimulatory molecules to reduce effector T‐cell activation [Color figure can be viewed at wileyonlinelibrary.com]

It has become increasingly clear that a strong selection process acts on cancer cell populations during the development of malignancy and antitumor treatment responses, and that this selection process regulates the tumor cell variants that escape tumor‐directed immune responses. Mechanisms that allow tumors to escape include reduced expression of costimulatory molecules, alterations in their MHC‐I expression, deficiencies in their antigen processing machinery, and other changes that can promote immune tolerance. Exosomes derived from both immune cells and tumor cells have been shown to carry tumor‐derived factors, indicating that TDEs may play an important role in intercellular communication between the tumor and the innate and adaptive immune systems. Several observations indicating that TDEs may mediate immune selection (Figure 3) are discussed below.

#### 3.5.1 TDE expression of the nonclassical MHC‐I protein HLA‐G

HLA‐G, a potent suppressive molecule that impairs effector functions of immune cells, has been detected in TDEs from a melanoma cell line,^122^ and ascites and pleural exudates derived from cancer patients.^170^ A recent study has demonstrated that TDEs derived from renal CSCs vs non‐CSCs, are the major mediator of an inhibitory effect on monocyte differentiation and maturation to DCs^171^ (Figure 3), and that this effect was mediated by HLA‐G^+^ TDEs. In this study and others,^123^ elevated TDE HLA‐G expression was associated with CSC level, both in vivo and ex vivo. High levels of HLA‐G^+^ TDEs in peripheral blood samples of breast cancer patients receiving neoadjuvant chemotherapy were also associated with disease progression, whereas high levels of soluble HLA‐G were associated with improved clinical outcomes. Notably, HLA‐G can be transferred from TDEs to HLA‐G^−^ exosomes.^123^ TDE‐mediated HLA‐G transfer also represents a potential mechanism through which HLA‐G^−^ target cells, including tumor cells, can acquire HLA‐G expression to promote their immune tolerance^123^ (Figure 3).

#### 3.5.2 TDE‐mediated suppression of MHC‐associated costimulatory molecules

In the presence of TDEs derived from melanoma cell lines, human DCs fail to upregulate costimulatory molecules, such as CD80 and CD86; paralleled by a significant release of inhibitory cytokines.^112^, ^113^ A comparable effect was also observed in exosomes isolated from the plasma of advanced melanoma patients, but not those isolated from healthy donors.^113^

#### 3.5.3 Regulation of T‐cell activity

Expression of the T‐cell receptor (TCR) and its associated signal transducing CD3‐ζ chain is important for the functional integrity of the signaling pathway in T‐cells.^172^ TDE exposure inhibits TCR expression and signaling^150^ and induces the release of inhibitory cytokines,^119^ leading to inadequate T‐cell activation and T‐cell apoptosis. TDEs can selectively inhibit activated CD8^+^ T‐cells by reducing their expression of CD3‐ζ and phosphorylated signal transducer and activator of transcription 5 (pSTAT5), to increase their apoptosis rate.^173^ Conversely, TDEs can exert the opposite effect on activated CD4^+^ T‐cells by increasing CD3‐ζ and pSTAT5 expression. These results derive from studies performed with TDEs from head and neck cancer and melanoma. Additional studies should be performed to address the potential conservation of the mechanism among different tumor types, but it is evident that TDE‐induced tolerance plays an important role in the ability of tumors to escape the immune response.

Taken together, the listed TDE‐mediated effects that favor the development of immune tolerance responses, suggest that TDEs may be one of the driving forces that render tumor cells resistant to CTL attack.

### 3.6 TDEs in the tumor metastasis

As discussed above, TDEs can exert regulatory influences on immune responses through both local and systemic effects, and can carry factors that stimulate or suppress immune responses. However, perhaps the most important systemic role of TDEs appears to reside in their ability to build new niches that facilitate the development of tumor metastasis.^174^ TDEs derived from metastatic melanoma cells can enhance metastasis formation by “educating” bone marrow progenitors and preparing a suitable niche to assist subsequent metastasis.^9^ This concept differs from the conventional “seed and soil” hypothesis of metastasis, which proposed that metastatic tumors develop only at sites where circulating cancer cells encounter favorable growth conditions, by adding the concept that primary tumors promote the development of “premetastatic niches.”^175^ It was subsequently found that TDE integrins could be used to predict organ‐specific metastasis and that targeting the integrins α6β4 (associated with lung metastasis) and αvβ5 (associated with liver metastasis) decreased and lung and liver TDE uptake and metastasis, respectively.^126^ Another study found that the uptake of pancreatic ductal adenocarcinoma TDEs by hepatic Kupffer cells increased their TGF‐β secretion to upregulate fibronectin production by hepatic stellate cells.^127^ Blocking TDE‐expressed macrophage migration inhibitory factor (MIF) prevented liver premetastatic niche formation and metastasis,^127^ Exosome expression of MIF could also differentiate patients with pancreatic tumors that did not progress from patients with stage I pancreatic ductal adenocarcinomas. These findings supported the critical role that TDEs play in the construction of premetastatic niches for tumor metastasis.^127^

## 4 REVERSING IMMUNE ESCAPE BY TDE‐TARGETING STRATEGIES

### 4.1 Reversing TDE‐mediated immune cell apoptosis

As previously discussed, the secretion of TDEs allow tumor cells to promote the apoptosis of activated T‐cells and NK cells to limit the ability of the immune system to attenuate tumor growth and progression by the depletion of tumor‐directed effector immune cells.^153^, ^176^ The depletion of these immune cells by TDEs can be blocked in experimental systems by treatment with mAbs directed against TDE proteins implicated in this regulation, including MHC‐I, Fas, Tim‐3, and galectin‐9.^116^, ^147^, ^153^ However, while these preliminary results are promising, extensive investigation is still required to determine the best choice for specific targets to attenuate TDE‐mediated apoptosis of tumor regulatory immune cells, and to validate their in vivo effectiveness and safety.

### 4.2 Neutralizing TDE‐mediated attenuation of Treg responses

**Figure 4:**
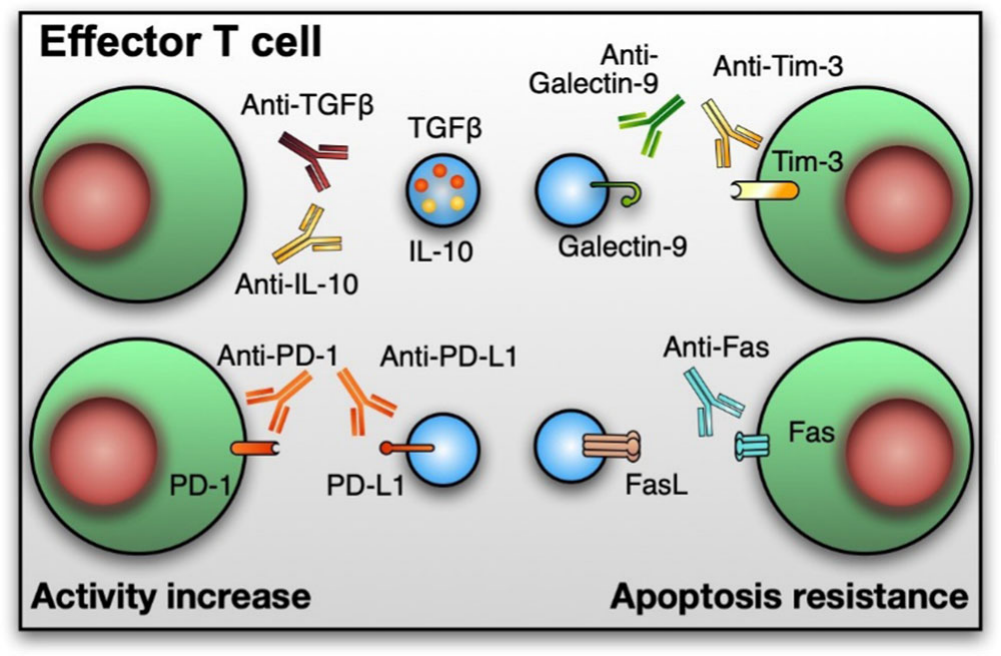
Strategies for targeting tumor‐derived exosome (TDE)‐mediated suppressive effects on effector T cells. Specific antibodies directed against TDE‐derived transforming growth factor‐β (TGF‐β), interleukin 10 (IL‐10), and programmed cell death ligand 1 (PD‐L1) or cell‐derived PD‐1 are being studied for their ability to block the suppressive TDE effects and promote effector T‐cell activation. Specific antibodies against TDE‐derived Galectin‐9 and Fas ligand (FasL) and cell‐derived Tim‐3 and Fas are being investigated for their potential to block TDE‐mediated apoptosis of effector T cells [Color figure can be viewed at wileyonlinelibrary.com]

Monitoring Treg responses in cancer patients shows promise as a means to improve traditional cancer therapies and new cancer vaccines, as well to evaluate the potential for the development of recurrent disease. Treg frequency and suppressor function are significantly higher in patients receiving oncologic therapies than in those with active disease,^177^ suggesting that inflammatory cytokines or other immune suppressive factors, including TDEs, are released by the tumor during antitumor treatment. Notably, antibody‐mediated neutralization of TDE‐associated TGF‐β1 and/or IL‐10 inhibited Treg induction and expansion during tumor therapy.^119^ A therapeutic strategy that consists of both a conventional therapy and a TDE‐targeted therapy may therefore represent a promising approach to induce more effective tumor‐directed immune responses, and thereby limit the potential for tumor recurrence and metastasis. There are now several potential targets for TDE‐targeted immunotherapy approaches to derepress immune function and improve tumor control (Figure 4).

### 4.3 TDE‐directed cancer vaccine approaches

Tumor‐specific immune responses can be induced by DC‐derived exosomes (DEX) after the DCs have been pulsed with tumor antigens^178^ or by using TDEs from tumor cells as a source of shared tumor rejection antigens for CTL cross‐priming.^110^, ^128^ Two phase I clinical trials have shown that it is possible to produce a DEX vaccine using DCs loaded with autologous melanoma‐associated antigen (MAGE) antigens and that DEX‐mediated vaccine therapies are well‐tolerated in patients with advanced MAGE^+^ melanomas^179^ and with advanced MAGE^+^ non–small‐cell lung cancer (NSCLC) tumors.^179^ Intriguingly, some of the immunized patients demonstrated stable disease.^179^ However, MAGE‐specific T‐cell responses were not detectable in peripheral blood samples from these patients,^180^ or minimal increases in peptide‐specific T‐cell activity were detected^179^ with enhanced NK cell effector functions. To improve the limited DEX‐induced T‐cell response, a “second generation” of DEXs has been developed that uses exosomes derived from DCs after their maturation by exposure to a TLR4 ligand or IFN‐γ. DEXs generated by such mature DCs have shown the ability to induce greater T‐cell stimulation than DEXs from immature DCs.^181^, ^182^, ^183^ In a recent phase II clinical trial, such second generation DEXs (IFNγ‐DEX) were loaded with MHC‐I and MHC‐II‐restricted tumor antigens and administered as maintenance immunotherapy to patients bearing inoperable NSCLC tumors after induction chemotherapy.^184^ The IFNγ‐DEXs did not induce antigen‐specific T‐cell responses, but successfully boosted the NK cell antitumor immunity. This enhancement of NK cell function correlated with prolonged PFS and with the IFN‐γ‐DEX content of MHC class II molecules and with DEX abundance of MHC‐II and the NKp30 ligand BAG6. These results suggest that DEXs may represent a cancer immunotherapy approach that operates by stimulating tumor‐specific NK cell activity.

TDEs also demonstrate the ability to induce protective antitumor immunity and tumor‐specific T‐cell responses in vitro and in an in vivo animal model.^110^, ^129^ However, pure TDEs cannot induce strong antitumor activities and must be modified to do so. Several studies have now examined the efficacy of TDEs modified to enhance antitumor‐specific immune responses. For example, TDEs derived from heat‐stressed CEA‐positive tumor cells contained more HSPs and MHC‐I,^109^, ^124^ and superantigen‐modified TDEs were found to induce stronger immunogenic responses.^185^ Cytokine treatment also shows promise for eliciting improved antitumor TDE responses. TDEs derived from tumors genetically modified to express IL‐18, promote enhanced cytokine release, DC cell maturation, and DCs pulsed with IL‐18^+^ TDEs induce more potent specific CTL responses than TDEs derived from cells without this IL‐18 modification.^186^ Chemokine‐containing TDEs from heat‐stressed cells also revealed a more efficient antitumor immune response.^135^ In one phase I clinical trial, patients with advanced colorectal cancer received subcutaneous immunizations with ascites‐derived exosomes (AEX) from ovarian carcinoma or AEX plus granulocyte‐macrophage colony‐stimulating factor (GM‐CSF).^187^ This AEX vaccine was determined to contain CEA, MHC proteins and HSPs, stimulated CTL responses, and was judged to be safe and tolerable, although only AEX plus GM‐CSF, but not AEX alone, showed a strong antitumor specific CTL response.

For both the DEX and TDE vaccines the effort to improve their immunotherapeutic action should focus on enhancing the visibility of their presented to the immune system as a target for CTL and NK responses. While substantial research much still be conducted to determine if cancer vaccines based on DEXs or modified TDEs will be safe and effective, the initial results offer some encouragement. One critical concern for the potential of these approaches, however, arises from the fact that exosomes carry a broad array of factors that can influence cell phenotypes and great care must be taken to reduce the potential that such exosome‐based vaccine approaches do not also enhance the potential for subsequent or concurrent tumor metastasis by promoting the formation of premetastatic niches. This concern is exacerbated by our limited understanding of the systemic effects of specific exosome populations and the processes and underlying mechanisms that influence these effects. Given that most exosome vaccine studies have not achieved significant success and carry poorly understood risks, it may be premature to address the clinical potential of such approaches.

## 5 CONCLUDING REMARKS AND FUTURE PERSPECTIVE

TDEs released from tumor cells not only alter the phenotype of adjacent normal cells and tumor‐infiltrating immune cells in the tumor microenvironment but also enter the systemic circulation to contact and alter the phenotype of distant cells, promoting the formation of distant premetastatic niches and altering the systemic immune repertoire to promote cancer development and metastasis. Mounting evidence indicates that changes in cancer cells and the tumor microenvironment can regulate the biogenesis, composition, and function of TDEs, however relative little is known about many of these processes, which could offer new avenues for tumor interventions. Indeed, at least one recent study has utilized a high‐throughput screening approach to identify the potential of repurposed drugs to act as selective inhibitors of exosome biogenesis.^188^ This approach represents a tempting means to limit the negative local and systemic effects of TDEs on tumor development and metastasis, including their effects on the antitumor immune response. However, since all cells appear to secrete exosomes, this type of intervention appears likely to cause significant side‐effects unless drugs can be identified that target tumor‐selective mechanisms regulating exosome biogenesis.

The tumor microenvironment regulates TDE production, immune cell infiltration, survival and activity, metastasis and chemotherapy resistance. Therapeutics that target tumor‐specific pathways that regulate these mechanisms offer another approach for cancer interventions. Most current research, however, has focused on employing TDEs in vaccine approaches designed to restore tumor‐specific immunity and thus promote tumor clearance. Such methods appear promising and have entered clinical trials, however, due to the potential of TDEs to inhibit antitumor responses and promote metastasis, substantial research needs to be performed to address safety concerns. Better insight into the interface between TDEs and innate and adaptive immunity could also provide novel tools to enhance antitumor responses. The heterogeneous origin and variable effects of TDEs represent a major challenge for future therapies aiming at silencing or modifying TDE effects on the immune system to promote tumor clearance. Substantial basic research and several clinical studies still need to be performed before currently proposed TDE‐based or TDE‐targeted therapies can be translated into clinical practice.

Significant progress has been made in understanding the mechanisms that control exosome biogenesis, although most of this effort has focused on the study of factors and processes associated with ESCRT‐dependent exosome secretion. Much less is known about ESCRT‐independent exosome production, or how either process is regulated in response to environmental or malignant changes known to increase exosome secretion, or how these changes affect selective enrichment of TDE cargoes to mediate their activities. Better understanding of these topics are urgently required to permit the development of target approaches that can attenuate TDE secretion or TDE‐mediated systemic activities, while sparing potentially critical exosome activities regulated by nonmalignant tissues. Future therapeutics that attenuate TDE‐mediated effects appear likely to require targeted strategies to attenuate TDE secretion or selectively block the packaging of factors involved in key TDE‐mediated effects on tumor development or metastasis. The rapid growth in our knowledge of how exosome biogenesis and how TDEs differ from exosome produced by nonmalignant cells and tissues, offers hope that the discovery of candidate therapeutic TDE targets may be achievable within the near future.
